# Evaluation of the Family Integrated Care model of neonatal intensive care: a cluster randomized controlled trial in Canada and Australia

**DOI:** 10.1186/s12887-015-0527-0

**Published:** 2015-12-15

**Authors:** Karel O’Brien, Marianne Bracht, Kate Robson, Xiang Y. Ye, Lucia Mirea, Melinda Cruz, Eugene Ng, Luis Monterrosa, Amuchou Soraisham, Ruben Alvaro, Michael Narvey, Orlando Da Silva, Kei Lui, William Tarnow-Mordi, Shoo K. Lee

**Affiliations:** 1 Maternal-Infant Care Research Centre, Mount Sinai Hospital, Toronto, ON Canada; 2 Department of Paediatrics, University of Toronto, Toronto, ON Canada; 3 Department of Paediatrics, Mount Sinai Hospital, 600 University Avenue Rm 19-231A, Toronto, ON M5G 1X5 Canada; 4 Women and Babies Program, Sunnybrook Health Sciences Centre, Toronto, ON Canada; 5 Dalla Lana School of Public Health, University of Toronto, Toronto, ON Canada; 6 Miracle Babies Foundation, Chipping Norton, NSW Australia; 7 Department of Pediatrics, Neonatal Division, Dalhousie University, Halifax, NS Canada; 8 Department of Pediatrics, University of Calgary, Calgary, AB Canada; 9 Department of Pediatrics and Child Health, University of Manitoba, Winnipeg, MB Canada; 10 Department of Paediatrics, Western University, London, ON Canada; 11 Department of Newborn Care, Royal Hospital for Women and Faculty of Medicine, University of New South Wales, Sydney, Australia; 12 WINNER Centre for Newborn Research, NHMRC Clinical Trials Centre, University of Sydney, Sydney, Australia; 13 Department of Infectious Diseases, Westmead Hospital, University of Sydney, Sydney, Australia

**Keywords:** Family-centered care, Family-integrated care, Infant, Premature, Neonatal intensive care unit

## Abstract

**Background:**

Admission to the neonatal intensive care unit (NICU) may disrupt parent-infant interaction with adverse consequences for infants and their families. Several family-centered care programs promote parent-infant interaction in the NICU; however, all of these retain the premise that health-care professionals should provide most of the infant’s care. Parents play a mainly supportive role in the NICU and continue to feel anxious and unprepared to care for their infant after discharge. In the Family Integrated Care (FICare) model, parents provide all except the most advanced medical care for their infants with support from the medical team. Our hypothesis is that infants whose families complete the FICare program will have greater weight gain and better clinical and parental outcomes compared with infants provided with standard NICU care.

**Methods/Design:**

FICare is being evaluated in a cluster randomized controlled trial among infants born at ≤ 33 weeks’ gestation admitted to 19 Canadian, 6 Australian, and 1 New Zealand tertiary-level NICU. Trial enrollment began in April, 2013, with a target sample size of 675 infants in each arm, to be completed by August, 2015. Participating sites were stratified by country, and by NICU size within Canada, for randomization to either the FICare intervention or control arm. In intervention sites, parents are taught how to provide most of their infant’s care and supported by nursing staff, veteran parents, a program coordinator, and education sessions. In control sites standard NICU care is provided. The primary outcome is infants’ weight gain at 21 days after enrollment, which will be compared between the FICare and control groups using Student’s *t*-test adjusted for site-level clustering, and multi-level hierarchical models accounting for both clustering and potential confounders. Similar analyses will examine secondary outcomes including breastfeeding, clinical outcomes, safety, parental stress and anxiety, and resource use. The trial was designed, is being conducted, and will be reported according to the CONSORT 2010 guidelines for cluster randomized controlled trials.

**Discussion:**

By evaluating the impact of integrating parents into the care of their infant in the NICU, this trial may transform the delivery of neonatal care.

**Trial registration:**

NCT01852695, registered December 19, 2012

## Background

### Background and rationale

In the highly technological environment of the neonatal intensive care unit (NICU), infants are physically, psychologically, and emotionally separated from their parents. Recognition that this experience impedes parent-infant interaction and is detrimental to the infant, led to the development of programs such as family-centered care, kangaroo care, and skin-to-skin care [1–3]. However, these programs are based on the common premise that only NICU professionals with special skills can provide the majority of care for the infant. Parents remain relegated to a supportive role, and some have described themselves as voyeurs who are “allowed” to visit and hold their infants [4, 5]. Many feel anxious and unprepared to care for their infants after discharge [6, 7]. This is in stark contrast to the regular nursery, where care is provided by parents from birth.

In 1979, a shortage of NICU nurses in Estonia prompted Levin [8, 9] to implement a “humane” care model in which parents provide nursing care for the infant (except for respiratory care and administration of intravenous [IV] fluid and medication), while nurses provide teaching and guidance to parents. In a non-randomised, before-after comparison, this model was associated with a 37 % improvement in weight gain in the first 20 days of life [9]. This study contributed to the growing body of evidence suggesting that hospitalized infants may thrive best in a quiet environment, with good nutrition, and consistent love and care from their parents, but without excessive stimulation and handling [6, 10, 11]. As first proposed by Bowlby in 1951 [12], the quality and quantity of the interaction between infants and their parents is particularly important to this concept. During the acute phase of NICU care, a variety of studies have reported that maternal presence, specifically through stimuli provided by voice and breast milk odor, results in more stable physiological responses [13, 14], improved oral feeding [15–18], fewer critical events [13, 19], and shorter length of stay [17, 18] for preterm infants. In the longer term, strong, responsive parent-infant interaction has been associated with improved behavioural outcomes in preterm infants [20–22], a relationship that may be inhibited by poor parental mental well-being [23–26]. As such, encouraging parental presence in the NICU, and providing parents with education and support to reduce their stress levels and improve their knowledge and confidence, is essential to improve preterm infant outcomes.

Building on the evidence from the literature and direct observation of the program in Estonia, we developed the Family Integrated Care (FICare) model specifically for the current Canadian NICU environment, to completely integrate parents into the NICU care team. The principle of FICare is that in the NICU, families should be supported, educated, and empowered to provide as much of their infant’s care as they are able [27, 28]. The FICare program includes a parent education program [29], a nursing education program [30], peer-to-peer support from ‘veteran’ parents [31], and adaptation of the unit policies, procedures, and other infrastructure as necessary, to provide social, psychological, and physical supports that enable greater parent participation.

In a pilot study of the FICare program conducted at Mount Sinai Hospital, Toronto, 31 FICare infants were matched 1:2 with control infants (*n* = 62) based on gender, gestational age (± 2 weeks), birth weight (± 300 grams), age at enrollment, and length of stay following enrollment of ≥ 21 days. The rate of change in weight gain was significantly higher in FICare infants compared with control infants (*p* < 0.05). There was also a significant increase in the rate of breastfeeding at discharge (82.1 vs. 45.5 %, *p* < 0.05). The mean Parental Stress Scale: NICU (PSS:NICU [32]) score for FICare mothers was 3.06 ± 0.12 at enrolment, which decreased significantly to 2.30 ± 0.13 at discharge (*p* < 0.05) compared with control mothers, whose stress scores were not significantly reduced (3.25 ± 0.19 at admission, 2.99 ± 0.2 on discharge, *p* > 0.05). Feedback regarding program implementation from the parents and nurses was very positive [33].

### Hypothesis

The FICare pilot study suggested that the model is feasible and safe in a Canadian healthcare setting, and may decrease parental stress and improve infant weight gain as well as other neonatal outcomes. To evaluate the impact of FICare on neonatal and parental outcomes, we designed and initiated a multi-national, multi-center cluster randomized controlled trial. Our hypothesis is that infants whose families complete the FICare intervention will have improved weight gain and better clinical outcomes compared with infants who received standard care in NICUs randomized to the control arm of the trial.

## Methods/Design

### Trial design

Due to the nature of the intervention, which involves changes to unit-level provision of care and interaction between participants, blinding of participants or NICU staff is not possible. Therefore, to avoid contamination of patients in the control arm, the cluster randomized controlled trial design was selected, in which level 3 NICUs were randomized but the intervention was targeted and the outcomes measured at the individual level. Presently, our prospective multi-centre cluster randomized controlled trial is being conducted at 19 tertiary level Canadian, 6 Australian, and 1 New Zealand NICU. The trial was designed, is being conducted, and will be reported according to the CONSORT 2010 guidelines for cluster randomized controlled trials [34]. Mount Sinai Hospital, where the pilot study of FICare was conducted, was assigned *a priori* to the intervention arm. Randomization was stratified by country, and within Canada was stratified by NICU size according to the number of yearly eligible admissions: < 200 (10 Canadian sites) or ≥ 200 (9 Canadian sites) admissions. Randomization of sites was performed using a random number generator. Enrollment in the trial commenced on 1^st^ April, 2013 and will continue until the required sample size of 675 infants in each arm is reached, which is estimated to occur in August, 2015.

### Study participants

Eligible study infants include those who are: i) born at ≤ 33 weeks’ gestation; and ii) on no respiratory support or on low-level respiratory support (i.e., oxygen by cannula or mask, or non-invasive ventilation such as continuous positive airway pressure [CPAP], biphasic CPAP and nasal intermittent positive pressure ventilation). As most infants born at ≤ 33 weeks’ gestation are not discharged home until at least 36 weeks’ postmenstrual age, a minimum “dose” of 3 weeks of in-hospital intervention is ensured. An additional inclusion criterion at the intervention sites is that the primary caregiver parent must commit to spending a minimum of 6 h per day with her/his infant between the hours of 7 am and 8 pm to enable attendance at medical rounds and education sessions.

Infants excluded from the study are those who: i) are receiving palliative care; ii) have a major life-threatening congenital anomaly; iii) have a critical illness and are unlikely to survive; iv) are on a high level of respiratory support (mechanical ventilation, high-frequency oscillatory or jet ventilation, extra-corporeal membrane oxygenation); v) are scheduled for early transfer to another hospital; or vi) have parents with an inability to participate (e.g., health, family, social, or language issues that might inhibit their ability to integrate with the health-care team).

### Enrollment

Parental consent is being obtained from families of eligible infants at both the intervention and control sites. For NICUs randomized to the FICare intervention arm, the site program coordinator approaches parents of all potentially eligible infants soon after admission to the NICU to explain the study verbally and deliver an information leaflet detailing the purpose and process of the study, as well as any possible detrimental effects of participation. Parents are screened to determine if there are barriers to their participation in the trial, and informed of the FICare education sessions, which they may attend regardless of whether they participate in the trial. Parents are then approached for informed consent when their baby becomes eligible (i.e., stable on CPAP). Families are enrolled in the trial, after consent is obtained (Day 0). All families approached are recorded in a patient eligibility log regardless of actual participation. Infants whose parents decline to participate in the study receive standard care at that site.

For NICUs randomized to the control arm, the site program coordinator approaches the parents of all potentially eligible infants for consent to collect infant data and information on parental stress and anxiety. The families of eligible infants at control sites are not screened to determine whether they would be willing to spend at least 6 h per day in the NICU as per the FICare protocol. The infants of parents who consent are enrolled in the study once they meet the inclusion criteria, and continue to receive standard care.

### Participating sites and research ethics approval

Ethics approval for the trial was obtained from the research ethics boards (REBs) of each of the following participating hospitals: Centre Hospitalier Universitaire de Quebec-Laval (Comité d’éthique de la recherche du CHU de Québec), Centre Hospitalier Universitaire de Sherbrooke (Comité d’éthique de la recherche en santé chez l'humain du CHUS), Children’s & Women’s Health Centre of BC (UBC C&W REB), Foothills Medical Centre (Conjoint Health REB), Hamilton Health Sciences Centre (Hamilton Integrated REBd), IWK Health Centre (IWK-REB), Janeway Children’s Health Centre (Health Research Ethics Authority), Kingston General Hospital (Queen’s University Health Sciences and Affiliated Teaching Hospitals REB), London Health Sciences Centre (University of Western Ontario REB for Health Sciences Research Involving Human Subjects), Moncton Hospital (Horizon Health Network REB), Mount Sinai Hospital (Mount Sinai Hospital REB), Regina General Hospital (Regina Qu’Appelle Health Region REB), Royal University Hospital (University of Saskatchewan Biomedical REB), Saint John Regional Hospital (Horizon Health Network REB), St. Boniface General Hospital and Health Sciences Centre Winnipeg (University of Manitoba Health REB), Sunnybrook Health Sciences Centre (Sunnybrook REB), The Hospital for Sick Children (SickKids REB), Victoria General Hospital (UVic/VIHA Joint Research Ethics Sub-Committee), Windsor Regional Hospital (Windsor Regional Hospital REB), The Canberra Hospital (ACT Health Human Research Ethics Committee), Dunedin Hospital (Central Health and Disability Ethics Committee), Gold Coast Hospital (South Eastern Sydney Local Health District Human Research Ethics Committee), Liverpool Health Service (South Western Sydney Local Health District Research and Ethics Office), Royal Hospital for Women (South Eastern Sydney Local Health District Human Research Ethics Committee), Royal North Shore Hospital Women (South Eastern Sydney Local Health District Human Research Ethics Committee), and The Townsville Hospital (The Townsville Hospital Human Research Ethics Committee).

### Privacy and confidentiality

All data are collected on a regular basis throughout the duration of the trial according to standardized definitions. The de-identified data are transferred to the CNN Coordinating Centre at the Maternal-Infant Care Research Centre, Toronto for analysis. All data access and use complies with the Health Information Act and the Personal Information Protection and Electronic Documents Act (PIPEDA) in Canada, the Privacy Act 1988 Sections 95 and 95A in Australia, and the Privacy Act 1993 and Health Information Privacy Code in New Zealand. Data security is compliant with standards established by the CNN and the Mount Sinai Hospital Research Ethics Board. Only de-identified information will be used in the analysis and publication of results. Publications will only use aggregate data.

### Patient withdrawals

Parents can withdraw themselves from the study at any time on their own request. If at any time it is identified that a parent is having difficulty taking on their new role or is feeling very stressed, the physician taking care of the infant will meet with the parent to see what additional supports are needed. Parents also have access to peer-to-peer support from veteran parents, social work support, and psychiatric consultation on an as-needed basis. If it is felt by the care team that it is not in the parent’s best interest to continue with the FICare model of care, this will be discussed with the parent and other options explored. If there is any identified risk to the infant by the parent’s continued participation, standard care will be applied. If an infant’s medical condition deteriorates such that he/she needs ventilation, or can no longer be provided adequate medical care in this model, the family’s involvement will be modified until the infant’s condition improves and they can resume full involvement.

### Intervention

Enrolled parents are oriented to the unit by a specially trained FICare program coordinator, who guides the parents in accessing the tools necessary for their self-education, and provides information on the charting/diary entries required and how they will be asked to assume responsibility for more of their infant’s care. Parents are expected to attend daily medical rounds, do basic infant charting, and maintain a diary to the best of their ability with the aim of providing them with greater knowledge of their infant’s medical status. Nursing support enables parents to provide care for their infant(s) through activities such as feeding, bathing, dressing, and holding skin to skin. Additional support, particularly around coping within the NICU, is provided to the parents by volunteer veteran parents [35] and through special education sessions (see *‘**Parent education program**’* below).

#### Resources

Resources are provided to facilitate parents’ ability to spend as much time as possible at the intervention sites. Each unit provides a lounge and sleep room for the exclusive use of parents, as well as amenities to facilitate parents spending extended periods of time in the hospital. Comfortable reclining chairs are provided in the NICU for parents to provide kangaroo and skin-to-skin care, while still being able to interact with other parents and staff, and breast pumps are available to facilitate breast feeding. Parents are also provided with subsidized parking or public transport vouchers.

#### Provider/nurse and parent volunteer education program

All doctors, nurses, respiratory therapists, social workers, and veteran parent volunteers at NICUs in the intervention arm have been trained in FICare. A team consisting of a neonatologist and the FICare program coordinator from each Canadian NICU attended a 2-day training program in Toronto. The training of the Australian units were conducted by the Toronto group in Sydney, Australia, while material from the Toronto group was used to construct a training workshop for the New Zealand unit in Dunedin, New Zealand. Training was provided by a multi-disciplinary team (neonatologist, nurse, psychologist, social worker, veteran parent volunteer) from Mount Sinai Hospital with experience in training staff gained during the pilot study. The training program was designed to provide the skills needed to teach other staff the concepts of FICare, including improving parent-infant interaction, re-conceptualization of the nursing role, coaching skills, psychological implications of preterm birth on parents, infant development, and discussions about life as a FICare nurse and a day in the life of a NICU parent [30]. Following this workshop, each NICU team organised training for their nursing staff, equivalent to a 4-h training workshop. Veteran parent volunteers were also orientated and trained within their own hospitals. Physicians and other health professionals were trained through presentations at existing staff meetings, rounds, and journal club-style forums.

#### Parent education program

##### Parent education sessions

At the intervention sites, a parent education program is provided with small group sessions three to five times per week. These sessions provide parents with information about the medical care of preterm infants, preterm newborn development, coping within the NICU, preparation for discharge, and how they can interact with their infant more effectively [29]. The sessions follow a three-week schedule but the content and timing of the sessions are adapted to the enrolled families’ needs. The sessions are led by the FICare program coordinator or a healthcare professional with expertise in the topic being discussed (e.g., lactation consultant, dietician, pharmacist, respiratory therapist, mental-health professional). Appropriate educational materials including handouts and reference material may also be provided. Information provided in the education sessions is reinforced at the bedside by nursing staff.

##### Parent checklist

Parents are also provided with a skills checklist to guide them and help them track their education and skill development. The checklist is used to evaluate parents’ progress throughout the program and make appropriate changes to the support provided as required.

##### Charting

Parents are expected to complete a chart for their infant on a daily basis including recording the infant’s activity, feeds, and output. They are also encouraged to keep a diary, which facilitates parental recall of special events with their infant. Both the parental chart and diary are used for communication during medical rounds, but are not part of the official medical record. Parents record their time spent at the bedside, performance of skin-to-skin care, and attendance at the education sessions. The primary nurse for each infant continues to complete the official medical record as per hospital protocol.

#### Psychosocial support

Parent-to-parent support plays a large role in the FICare model of care. The physical clustering of FICare families together in the NICU and their participation in small group education sessions facilitate interaction and sharing of experiences. Volunteer veteran parents, who have had prior experience of having an infant admitted to the NICU, visit each NICU, organize recreational activities, and provide telephone support for families. This peer-to-peer support system aims to develop a sense of community among the FICare families [31]. Additional supports such as social work and psychiatric consultation are provided on an as-needed basis.

### Data collection

Data collection at both the intervention and control sites commences at enrollment and continues for the trial duration (21 days) and through to discharge from the NICU. Data collection utilizes the existing CNN database platform [36] in Canada, and in Australia and New Zealand, the Australian and New Zealand Neonatal Network (ANZNN) data system [37]. Data collected include demographics, antenatal and obstetric risks, delivery complications, admission illness severity scores, and selected practices and outcomes related to this trial. At each site, a trained research assistant abstracts data daily from patient charts directly into a laptop computer using a customized data entry program with built-in error checking and a standard manual of definitions. SSL-encrypted data are electronically transmitted directly from the Canadian sites to the CNN Coordinating Centre for verification and further cleaning. The Australia and New Zealand sites collate data via the ANZNN data verification system and submit encrypted data in batches to the CNN Coordinating Center.

Questionnaires and surveys are administered to parents by site program coordinators, and are available online and on paper. Data entered into online surveys are automatically included in a survey database and answers from paper documents are entered into the database by the program coordinator. In addition, in the Australia and New Zealand sites parents have the preferred option of entering answers directly via smartphone to a purpose-built web-based dataset that is being collated at the ANZNN Coordinating Centre.

### Outcomes and measures

The primary outcome of the trial is weight gain at 21 days since enrollment in the program, as measured by the z-score [38]. The z-score refers to the exact number of standard deviations greater or smaller than the median, and is used to monitor the growth of the infant relative to the expected intrauterine growth rate. It is standardized to population growth standards and superior to percentiles for infants whose size is outside of the normal range of a growth chart. As part of standard NICU practice infants are weighed at the same time each day with their diaper removed. Many infants are weighed on special scales built into their incubator. To decrease any risk of measurement bias nurses/parents are asked to first recalibrate the scales, then weigh the infant three times, and take the average weight. The bedside nurse charts the infant’s weight as per usual practice.

The secondary outcomes are: i) weight gain velocity at 21 days since enrollment and weight gain velocity from birth to 36 weeks corrected gestational age; ii) parent stress and anxiety; iii) breastfeeding rate at hospital discharge; iv) clinical outcomes including NICU mortality and major neonatal morbidities; v) safety as indicated by the number of critical incident reports per 1000 patient days; and vi) resource use including duration of oxygen therapy, duration of hospital stay, and potential cost savings due to reduced length of stay estimated using per diem costs [39].

The major neonatal morbidities include ≥ stage 2 necrotizing enterocolitis (NEC) defined according to Bell’s criteria [40]; bronchopulmonary dysplasia (BPD) defined as oxygen dependency at 36 weeks postmenstrual age or at the time of transfer to a level 2 centre [41]; nosocomial infection (NI) defined using the Center for Disease Control criteria [42]; ≥ stage 3 retinopathy of prematurity (ROP) classified according to the International Classification [43]; and ≥ grade 3 intraventricular hemorrhage (IVH) defined according to the criteria of Papile et al. [44] from cranial ultrasound during the first 28 days of life.

In both the intervention and control sites, parental stress and anxiety are measured using questionnaires (PSS:NICU and the State Trait Anxiety Index [STAI]) administered to parents at enrollment (Day 0) and Day 21 following enrollment. The PSS:NICU is a validated instrument to measure parents’ perceptions of stress within the NICU [32]. It comprises a 46-item self-report instrument that consists of four subscales that measure stress related to the: a) sights and sounds of the unit, b) appearance and behaviour of the infant, c) impact of the parents’ role and their relationship with their infant, and d) parents’ relationship and communication with the staff. The STAI is the definitive instrument for measuring anxiety in adults [45]. It is well validated, simple to use, and available in 40 languages. The STAI Form Y comprises of 40 items that can be completed in about 10 min, and measures state and trait anxiety. It provides a measure of the severity of the overall anxiety level.

### Sample size calculation

The proposed sample size of 675 infants in each arm of the trial was estimated for the primary outcome of weight gain at 21 days post-enrollment, as measured by the change in z-score (z-score at Day 21 – z-score at enrollment), using preliminary data from the FICare pilot study available at the time of trial design. These data included 20 infants in the FICare group with mean change in z-scores of 0.58 (standard deviation of 0.57), and 40 matched controls with mean change in z-scores of 0.42 (standard deviation of 0.43). Based on this result (38 % increase in mean z-score change) and Levin’s study [9], we anticipated at least a 25 % increase in mean z-score change in the FICare group. The sample size was estimated assuming the above standard deviation estimates from the preliminary data, and using Kerry’s method [46, 47] for unequal cluster sizes, given that 16 sites (6 large with average size of 315 eligible infants per year, and 10 small with average size of 113 eligible infants per year) had agreed to participate in the trial at that time. The sample size of 675 infants per arm has 80 % power to detect a ≥ 25 % difference (absolute difference of 0.11) in z-score change assuming a significance level of 0.05, intra-cluster correlation coefficient (ICC) of 0.01 [48, 49], and a 10 % drop-out rate.

The estimated sample size is feasible to achieve given that approximately 3200 eligible infants are admitted each year to the 16 sites committed at the time of trial design. We also note that statistical power and the number of eligible infants has been increased by recruitment of additional sites; in total 19 Canadian sites (10 large sites and 9 small sites) and 7 Australian/New Zealand sites (all small) have been randomized and approximately 4300 infants are eligible for enrollment annually.

### Statistical analysis

The unit of analysis will be the individual infant, and all analyses will be based on the intention to treat principle. The distribution of baseline characteristics in the study population will be summarized at the individual and cluster level within each FICare and control groups, using descriptive statistical methods. The primary outcome of weight gain at 21 days post enrollment, will be compared between the FICare and control groups using Student’s *t*-test adjusted for the inflation factor (or design effect) with a minimum variation weight correction [46, 47] to account for intra-cluster correlation and imbalance of cluster sizes. In addition, a two-level hierarchical linear regression model will examine the primary outcome, accounting for clustering, and potential confounders including patient-level characteristics (birth weight, gestational age, small for gestational age, gender, multiple births, admission illness severity, caesarean section, chorioamnionitis, maternal hypertension or diabetes, maternal education, parity) and NICU-level covariates (NICU size, teaching institution).

Secondary outcomes will be compared between the FICare and control groups using similar methods including the Student’s *t*-test for continuous variables and the Chi-square test for categorical variables adjusted for clustering [50, 51], as well as hierarchical linear or logistic regression models, as appropriate. Furthermore, weight change over time will be examined longitudinally using multivariable multi-level hierarchical models to compare the rate of change in weight gain between infants from the two trial arms.

While we realize that multiple comparisons are a concern when more than one analysis of the data is performed, we are not interested in the joint confidence region for all of our hypotheses at once. Rather, we are interested in them one at a time. Under these conditions, Rothman and Greenland [52, 53] argue that “multiple inference procedures … are irrelevant, inappropriate and wasteful of information” because they produce improperly imprecise single intervals.

Prior to data unmasking and analyses, issues relating to missing data and potential sources of bias will be examined and appropriate correction methods determined.

### Limitations and feasibility

A cluster randomized trial design was selected to evaluate FICare as it is an organizational and behaviour intervention where blinding of participants or investigators is not possible. Cluster randomized controlled trials are more effective at preventing contamination, but more susceptible to biases than trials randomizing individual subjects [54, 55]. In this trial, possible allocation bias is being minimized by the use of appropriate study design methods for cluster randomization including stratification (sites within each country randomized separately; big and small sites in Canada randomized separately) with randomization performed using a random number generator.

Selection bias may arise due to prospective recruitment of families after randomization of NICU sites, and differences in the nature and process of obtaining informed consent. Study coordinators who recruit families are not blinded to their site’s allocation and families consent to participate in rather than be randomized to the FICare intervention or control protocol. At intervention sites, consent is given to participate in the FICare program and to complete study questionnaires and collect infant data, whereas, at control sites consent is given for questionnaire completion and data collection only. Notably, the inclusion criteria at FICare sites require a time commitment of ≥ 6 h, whereas families at control sites are not specifically asked if they would be able to make the same commitment. As such, participants at intervention sites who commit to FICare may differ from those at the control sites, who do not have to commit to spending extended time in the NICU. For example, families who enroll in FICare may be at a higher socioeconomic level or have greater family support than control families.

To assess selection bias, differences in the enrolment rate at FICare and control sites will be examined. Multivariable analyses will correct possible confounding bias by adjusting for baseline factors including social, economic (parental education, employment), demographic (maternal age, family size), obstetric (parity, pregnancy complications) and infant factors (gestational age, small for gestational age, illness severity at admission, and age at enrollment). Further analyses will consider propensity score methods accounting for imbalance of baseline characteristics between FICare and control groups [54]. While we will attempt to identify and adjust for all the possible confounding variables, we also acknowledge that any selection/participation bias could be due to some unmeasured element of “parent engagement” at the initiation of the intervention.

Subject attrition may also produce biased results and impact generalizability. Families in the FICare group are provided with as much physical, psychological and financial support as possible (including parent lounge; subsidized parking or a transit pass; access to peer-to-peer, social, and psychiatric support services), but no such supports are available to control families. Attrition bias will be minimized by performing statistical analyses according to the intention to treat principle. To minimize losses due to retro transfer from level 3 to level 2 units prior to completion of the 21-day trial period, the original study protocol has been amended to allow follow-up of these infants within level 2 units provided these units obtain Research Ethics Board approval, receive appropriate training, and comply with the study protocol including providing nurse education and parent education programs.

Possible bias due to differential withdrawal or study drop-out will be assessed by examining the distribution of baseline factors between families who consent and complete the study, and eligible families who consent and start but do not complete the study within the intervention and control sites. Furthermore, a sensitivity analysis will compare results from complete-case data and from all families who were enrolled where missing data are imputed using methods that account for clustering [56, 57].

Recognizing the limitations of the cluster randomized controlled trial design, we have planned to thoroughly examine factors that may confound the effect of FICare with infant and parental outcomes, to correct analyses for bias when feasible, and to report possible residual bias.

## Discussion

In this age of accountability, infant outcomes, parental mental health, and reduction of health care costs are important objectives. The FICare program addresses all these issues because it can potentially improve infant outcomes, decrease parental stress and anxiety, and reduce resource use including duration of oxygen therapy and length of hospital stay with potential per diem cost savings. Improvement of neonatal outcomes by FICare may lead to reduced mortality and morbidity post-NICU discharge. In addition, FICare aims to increase the confidence and capability of parents to care for fragile preterm infants when they go home, which may reduce the need for post-discharge support for families, outpatient clinic visits, re-hospitalizations, and other health care utilization. Future studies are required to examine the longer-term effects of FICare. Additional trials may be conducted to assess the feasibility, safety, and efficacy of expanding the FICare model of care to NICU infants who are more acutely ill, such as those on a mechanical ventilator or who require surgery. If effective, the FICare model could represent a paradigm shift in approach to health care that may be applicable to other areas such as pediatrics, palliative care, geriatrics and chronic care.

## Abbreviations

BPD: bronchopulmonary dysplasia
CNN: Canadian Neonatal Network
CPAP: continuous positive airway pressure
FICare: Family Integrated Care
ICC: intra-cluster correlation coefficient
IVH: intraventricular hemorrhage
NEC: necrotizing enterocolitis
NI: nosocomial infection
NICU: neonatal intensive care unit
PIPEDA: Personal Information Protection and Electronic Documents Act
PSS:NICU: Parental Stress Survey: Neonatal Intensive Care Unit
ROP: retinopathy of prematurity
STAI: State Trait Anxiety Index
